# Carbapenem-Resistant but Cephalosporin-Susceptible *Pseudomonas aeruginosa* in Urinary Tract Infections: Opportunity for Colistin Sparing

**DOI:** 10.3390/antibiotics9040153

**Published:** 2020-04-01

**Authors:** Márió Gajdács

**Affiliations:** 1 Department of Pharmacodynamics and Biopharmacy, Faculty of Pharmacy, University of Szeged, 6720 Szeged, Hungary; gajdacs.mario@pharm.u-szeged.hu; Tel.: +36-62-341-330; 2 Institute of Clinical Microbiology, Faculty of Medicine, University of Szeged, 6725 Szeged, Hungary

**Keywords:** *Pseudomonas aeruginosa*, urinary tract infection, carbapenem, cephalosporin, resistance, uncommon phenotype, phenotypic, efflux pump, ampicillin C (AmpC)

## Abstract

This paper briefly reports the occurrence and epidemiology of carbapenem-resistant but cephalosporin-susceptible (Car-R/Ceph-S) *Pseudomonas aeruginosa* isolates from urinary tract infections (UTIs) in a tertiary-care hospital in the Southern Region of Hungary, and the phenotypic characterization of the possible resistance mechanisms in these isolates. Isolates and data were collected regarding *P. aeruginosa* UTIs corresponding to the period between 2008 and 2017. Susceptibility testing was performed using the Kirby–Bauer disk diffusion method; minimum inhibitory concentrations (MICs) of the isolates were determined using E-tests. The phenotypic detection of ampicillin C-type (AmpC) β-lactamases, efflux pump overexpression and carbapenemase production was also performed. *P. aeruginosa* represented *n* = 575 (2.72% ± 0.64%) from outpatient, and *n* = 1045 (5.43% ± 0.81%) from inpatient urinary samples, respectively. Based on the disk diffusion test, *n* = 359 (22.16%) were carbapenem-resistant; in addition to carbapenems, *n* = (64.34%) were also resistant to ciprofloxacin; *n* = (60.17%) to gentamicin/tobramycin; *n* = (58.51%) to levofloxacin; and *n* = (27.57%) to amikacin. From among the carbapenem-resistant isolates, *n* = 56 (15.59%) isolates were multidrug-resistant, while *n* = 16 (4.46%) were extensively drug-resistant. From among the Car-R/Ceph-S isolates (*n* = 57), overexpression of AmpC was observed in *n* = 7 cases (12.28%); carbapenemase production in *n* = 4 (7.02%); while overexpression of efflux pumps was present in *n* = 31 (54.39%) isolates. To spare last-resort agents, e.g., colistin, the use of broad-spectrum cephalosporins or safe, alternative agents should be considered in these infections.

## 1. Introduction

The emergence of drug resistance in Gram-negative bacteria is an increasingly severe public health issue, limiting the therapeutic armamentarium of clinicians [1,2]. From the standpoint of antimicrobial resistance (AMR), non-fermenting Gram-negative bacteria (NFGNB) are especially problematic due to the many intrinsic and acquired resistance mechanisms they possess or can acquire [3,4]. In addition, these pathogens are extremely adaptable to biologically unfavorable conditions, e.g., in harsh hospital environments; thus, they may become a source of opportunistic, nosocomial infections [5]. Urinary tract infections (UTIs) are the second most common infectious pathologies in developed countries, corresponding to an important economic and disease burden [6,7]. Previously, the treatment of urinary tract infections was relatively straightforward due to the predictable pathogen profile and resistance characteristics; however, with the emergence of AMR and the increasing number of elderly patients with underlying illnesses or immunosuppression, the treatment of UTIs is increasingly challenging in everyday clinical practice [8,9]. There is a growing appreciation for the clinical relevance of NFGNB in UTIs; among other organisms, the etiological role of *P. aeruginosa* has been highlighted by several studies worldwide, especially in debilitated patients or catheterized individuals [10,11]. *Pseudomonas* infections may be treated by utilizing β-lactams, fluoroquinolones, aminoglycosides and, in cases of extensive resistance, ceftolozan/tazobactam and colistin remain as unattractive (due to toxic adverse events or economic considerations), but viable, therapeutic alternatives [12]. β-lactam antibiotics may be considered as the backbone of antibiotic therapy, especially in vulnerable patient populations (e.g., children, pregnant women, the elderly) [13]. For *P. aeruginosa*, ceftazidime, cefepime (third and fourth generation cephalosporins, respectively) and carbapenems (imipenem, meropenem and doripenem) are relevant therapeutic choices [14]. Nevertheless, resistance in *Pseudomonas* to β-lactams may be due to a plethora of different resistance mechanisms: chromosomal production of ampicillin C-type (AmpC) β-lactamases, acquired cephalosporinases or carbapenemases, overexpression of efflux pumps, modifications in the penicillin-binding proteins (PBPs) and downregulation or absence of the OprD porin (i.e., porin mutants) [3,15]. Carbapenemase enzymes have the broadest spectrum of substrates (from penicillins to carbapenemases); they may eliminate β-lactam antibiotics as viable therapeutic options, depending on their substrate-profile. However, these enzymes are very diverse with variable spectra of activity and phenotypic expression levels [16,17]. Several studies have noted the occurrence of carbapenem-resistant but cephalosporin-susceptible (Car-R/Ceph-S) *P. aerugonisa* [14,18,19]. In these strains, the AmpC production is thought to be moderate or low, which would allow for the use of relevant cephalosporins, instead of other, more toxic agents with a disadvantageous side effect profile [20]. The aim of the present study was to investigate the occurrence and epidemiology of Car-R/Ceph-S *P. aeruginosa* isolates in a tertiary-care hospital in the Southern Region of Hungary, in addition to the phenotypic characterization of the possible resistance mechanisms in these isolates.

## 2. Results and Discussion 

### 2.1. Epidemiology and Antibiotic Susceptibility of Car-R/Ceph-S P. aerugonisa from UTIs

Between 2008 and 2017, *n* = 21,150 positive urine samples were received from outpatient clinics and *n* = 19,325 from inpatient departments. Among clinically significant pathogens overall, *P. aeruginosa* represented *n* = 575 (2.72% ± 0.64%) from outpatient, and *n* = 1045 (5.43% ± 0.81%) from inpatient samples, respectively (resulting in a sum of *n* = 1620 samples). Based on the disk diffusion test, *n* = 359 (22.16%) were carbapenem-resistant (Car-R, imipenem or meropenem minimum inhibitory concentration (MIC) ≥ 8 mg/L; *n* = 80 were imipenem-resistant and 96.25% were also resistant to meropenem in outpatient samples, while *n* = 279 were imipenem-resistant and 91.39% were also resistant to meropenem in outpatient samples, respectively; *p* = 0.016). The distribution of Car-R isolates was calculated from clinical samples taken from the following: first-stream urine: *n* = (2.22%); midstream urine: *n* = (27.57%); and catheter-specimen urine: *n* = (70.21%). The median age of patients affected by Car-R UTIs was 62 years (range: 0–90 years), with a female-to-male ratio of 0.59 (37.33% females); the majority of patients (59.71%) were aged 65 years or older. Some 22.28% of the isolates originated from outpatient clinics, while 56.69% came from the Intensive Care Departments (ICUs), 15.54% from the Dept. of Internal Medicine, and 5.49% from the Dept. of Traumatology.

In addition to carbapenems, *n* = 231 (64.34%) were also resistant to ciprofloxacin; *n* = 216 (60.17%) to gentamicin and tobramycin; *n* = 210 (58.51%) to levofloxacin; and *n* = 99 (27.57%) to amikacin, respectively. No colistin resistance was found among Car-R isolates. Resistance levels were significantly higher in inpatient isolates for ciprofloxacin (*p* = 0.031); levofloxacin (*p* = 0.04); and gentamicin/tobramycin (*p* = 0.038). From among the Car-R isolates, *n* = 56 (15.59%) isolates were multidrug-resistant (MDR), while *n* = 16 (4.46%) were extensively drug-resistant (XDR). 

Car-R/Ceph-S isolates were detected in *n* = 57 [100%] cases (*n* = 21 [36.84%] from outpatient samples and *n* = 36 [63.16%] from inpatients, respectively; *p* = 0.041). All isolates (100%) were ceftazidime-susceptible (100%; MIC ≤ 8 mg/L), while *n* = 43 (75.43%) isolates were cefepime-susceptible (MIC ≤ 8 mg/L). The MIC_50_ and MIC_90_ values of imipenem, meropenem, ceftazidime and cefepime were 16 and 64 mg/L; 8 and 32 mg/L; 2 and 8 mg/L; and 8 and 32 mg/L, respectively.

### 2.2. Phenotypic Characterization of Resistance Determinants in Car-R/Ceph-S P. aerugonisa Isolates

From among the Car-R/Ceph-S isolates (*n* = 57), overexpression of AmpC was observed in *n* = 7 (12.28%) cases (5 from inpatient samples, 2 from outpatient samples), exclusively in isolates that were susceptible to ceftazidime, but resistant to cefepime. A notable decrease in the ceftazidime MICs was seen in the presence of cloxacillin. The modified Hodge test was positive (indicating the presence of a carbapenemase) for *n* = 4 (7.02%) *P. aeruginosa* isolates.

Based on the phenotypic assay utilized in our study, overexpression of efflux pumps was present in *n* = 31 (54.39%) cases (25 from inpatient samples, 6 from outpatient samples). In *n* = 22 isolates (38.59%), decreases in the MICs in the presence of PAβN were observed for both imipenem and meropenem, while in *n* = 7 isolates (12.28%), the MIC decreased for imipenem, and in *n* = 2 cases, for meropenem only. Simultaneous occurrence of AmpC overexpression and efflux pump overexpression was shown in *n* = 5 (8.77%) cases, while simultaneous carbapenemase overexpression and efflux pump overexpression was detected in *n* = 2 (3.51%) isolates. No resistance determinants could be phenotypically verified in *n* = 15 (26.31%) isolates.

### 2.3. Discussion and Literature Review

*P. aeruginosa* is one of the most common opportunistic pathogens in hospital-acquired infections, associated with significant morbidity and mortality in the affected patients, especially in cases of infections caused by MDR/XDR isolates [21]. The aim of the present study was to demonstrate the occurrence of Car-R/Ceph-S *P. aeruginosa* from urinary tract infections over a 10-year surveillance period in a tertiary-care hospital in Hungary. During the study period, 57 such isolates were detected (corresponding to an average of 5–6 isolates/year), including 7 isolates with overexpression of AmpC β-lactamases, 4 isolates producing carbapenemase enzymes and 31 isolates with overexpressed efflux pumps, based on the phenotypic detection methods utilized. Efflux pump overexpression was shown to be the most common mechanism of resistance detected in this study, in addition to seven isolates where efflux pumps and β-lactamases were present simultaneously. No conclusive data could be obtained for the resistance determinants in fifteen isolates. Although it may be assumed that porin downregulation or deletion may be the underlying mechanism of resistance in these cases, no results from phenotypic or genotypic assays corroborate this hypothesis.

*P. aeruginosa*, as a urinary pathogen, has received substantial attention in recent years, which may be attributed to its increased prevalence in catheter-associated infections of hospitalized and/or immunocompromised patients [10,22]. In addition, recent studies have highlighted the most important virulence factors associated with *P. aeruginosa* in catheter-associated infections [23]. Epidemiological reports are scarce in this field; however, most studies available conclude that there is a significantly higher risk for inpatients and that debilitated patients older than 65 years of age are the most affected [23,24]. Over a three-year period, Ferreiro et al. recorded *n* = 62 nosocomial UTIs, with an overall 30-day mortality rate of 17.7% [24]. A Japanese study group reported 76 patients (*n* = 59 male and *n* = 17 female) with a *P. aeruginosa* UTI infection over a four-year period [25]. In a study from India, the prevalence of NFGNB (including *P. aeruginosa*) in UTIs was <2% [26]. As part of a national prospective surveillance study for nosocomial infections in ICUs in France, Venier et al. reported *n* = 525 (16% overall) *P. aeruginosa* UTIs; in addition, they identified male sex, previous antibiotic therapy, hospital admission and transfer from another ICU as individual risk factors [27]. Finally, in a study from Israel, Marcus et al. identified *n* = 28 (8% overall) community-acquired *P. aeruginosa* UTIs in children [28].

There are very few studies available in the literature reporting on the phenomenon of Car-R/Ceph-S *P. aeruginosa*, and most of these publications originate from the Far East. A Chinese study consisting of Car-R/Ceph-S *P. aeruginosa* collected between July and October 2011 found *n* = 29 individual isolates [19]. In this study, the authors demonstrated that decreased expression or deletion of the OprD porins was the most common mechanism of resistance (using Western blotting and RT-PCR), while production of carbapenemase or Ambler class C β-lactamase enzymes was not observed in these isolates. In another study by Chinese authors, *P. aeruginosa* isolates from bacteremic patients between 2010 and 2017 showing the Car-R/Ceph-S phenotype were assessed [29]: during the eight-year period, *n* = 63 isolates were detected and most of the microorganisms showed efflux pumo-overexpression and decreased expression of OprD. Similar to the previous study, production of relevant β-lactamase enzymes was not noted. In this report, the overall 30-day mortality rate of affected patients was also assessed, and was shown to be 27.0% [29]. In a Korean study, *n* = 18 ceftazidime-susceptible isolates were found in 77 imipenem-resistant *P. aeruginosa* samples [30]. In their report, thirteen isolates showed overexpression of the efflux pump genes mexB, mexD, mexF and mexY, and two isolates presented with ampC gene overexpression; all Car-R/Ceph-S isolates showed a significant decrease in oprD gene expression. All of the eighteen isolates were resistant to cefepime [30]. Khuntayaporn et al. described the characterization of *n* = 293 *P. aeruginosa* isolates collected between 2007 and 2009 [15]. Decreased oprD gene expression was observed in the majority of isolates (>93%), in addition to overexpression of efflux pumps in around two thirds (63.5%) of the isolates. Metallo-β-lactamase production and AmpC β-lactamase hyperproduction showed a lower prevalence (18.5% and 3.9%, respectively) [15]. A study from Brazil, spanning the years 2012 and 2013, reported *n* = 25 individual *P. aeruginosa* isolates from all clinical sample types [18]. Most of these bacteria had reduced oprD gene expression levels, while AmpC hyperproduction, carbapenem hydrolysis (detected by the MALDI-TOF MS method) and efflux pump overexpression (RT-PCR) were not shown in these isolates. A recently published clinical study from Israel (corresponding to the time period between 2010 and 2014) reported *n* = 67 monobacterial Car-R/Ceph-S bloodstream infections (mainly associated with UTIs and pneumonia), where the authors highlighted that cephalosporins were relevant therapeutic options for these infections [31]. In addition, a study from Iran identified *n* = 23 Car-R/Ceph-S *P. aeruginosa* isolates from 243 clinical samples, from the time period between 2016 and 2018 [14]. From among the 23 Car-R/Ceph-S samples, none of the isolates were positive for carbapenemase genes. Overexpression of AmpC was detected in *n* = 1 (4.3%) isolate. The phenotypic assay for overexpressed efflux pumps was positive in *n* = 14 (60.9%) isolates, while overexpression of relevant efflux pumps was shown in 47.3%–68.8% (depending on the efflux pump) by RT-PCR [14]. 

Carbapenems may be considered as the last line of safe therapeutic alternatives in many infections; however, carbapenem resistance is on the rise, both in NFGNB and in the members of the Enterobacterales order [11,16,32,33]. While in the latter group, Car-R is predominantly caused by the acquisition of plasmid-encoded carbapenemases, in NFGNB (including *Pseudomonas* spp.), there are several possible resistance mechanisms at play, including degrading enzymes, modifications in the transpeptidases, porin deletion and the overexpression of efflux transporters [2,3,4,10,12,14,19,34]. In addition, *Pseudomonas* species are members of the “SPACE” (*Serratia* spp., *Pseudomonas* spp., *Acinetobacter* spp., *Citrobacter* spp., *Enterobacter* spp.) organisms, characterized by inducible AmpC-based resistance [20,35]. Although the overlap between these resistance mechanisms may be significant, cases of Car-R/Ceph-S may be identified where the use of cephalosporins instead of colistin may be warranted [36]. Colistin is currently considered a last-resort, life-saving antibiotic for resistant Gram-negative infections [37]. Irrespective of its disadvantagous adverse effects and difficult dosing, based on pharmaco-epidemiological studies, the use of this agent is on the rise [38]. Nevertheless, its increased use will undeniably lead to increased resistance levels to this drug, as this phenomenon has been demonstrated for many other anti-pseudomonal antibiotics [39]. Car-R/Ceph-S isolates present an opportunity for microbiological/diagnostic stewardship, and a viable target for colistin-sparing stewardship interventions [40]. Although colistin resistance in *P. aeruginosa* is still relatively rare (especially in countries limiting the use of this agent), nevertheless, a steady increase in the resistance levels for colistin may be expected in the coming years [41]. Similar to the findings of other reports [14,18,19], resistance levels to other antibiotics were also pronounced, with ~60% resistance to fluoroquinolones and gentamicin. 

Limitations of the present study include the lack of molecular biological methods used to further characterize resistance determinants in the detected isolates, e.g., for the detection of various bla genes (bla_NDM_, bla_KPC_, bla_OXA-48_, and bla_VIM_, among others), and the detection of the overexpression of efflux pump genes (mexAB, mexCD, mexXY) or the reduced expression of the oprD porin compared to a housekeeping gene [14]. The prevalence of metallo-β-lactamase enzymes in Car-R *P. aeruginosa* isolates may be wide-ranging, based on literature findings (2%–100%) [42]. However, the hypothesis of Khalili et al. states that the occurrence of Car-R/Ceph-S isolates is due to the absence of carbapenemases, leading to the susceptible phenotype for ceftazidime and cefepime [14]. This may point to the relevance of OprD porin mutation as a substantial mechanism of resistance solely for carbapenems [15]. Although there are only a few studies available, the proportion of strains with reduced OprD expression or lack of OprD was very high (100% [19]; 100% [30]; 93.85% [15]; >90% [29]; 78.26% [14], and 41.38% [18], respectively). The role of OprD mutation in carbapenem resistance has been highlighted in repeated exposure to carbapenems [14,15,43]. In addition, this study was limited to isolates from UTI infections, while other studies included *P. aeruginosa* isolates from various clinical samples. Nevertheless, the present report is still relevant. 

## 3. Materials and Methods 

### 3.1. Study Design and Data Collection 

The present study was carried out using isolates and collected data, corresponding to the time period between January 1, 2008 and December 31, 2017, at the Institute of Clinical Microbiology, University of Szeged (Szeged, Hungary). The Institute serves as the dedicated microbiology laboratory of the Albert Szent-Györgyi Clinical Center, which is a 1820-bed primary- and tertiary-care teaching hospital, affiliated with the University of Szeged [44]. Data collection was performed electronically by searching the records of the laboratory information system (LIS) for urine samples positive for *P. aeruginosa*, based on the criteria below.

Samples with clinically significant colony counts for *P. aeruginosa* (>10^5^ CFU/mL) that were positive for nitrite and leukocyte-esterase tests were included; however, this was subject to interpretation by senior clinical microbiologists performing identification and reporting, based on the information provided on the clinical request forms for the microbiological analysis and international guidelines [10]. Only the first isolate per patient was included in the study; however, isolates with different antibiotic susceptibility patterns from the same patient were considered as different individual isolates. To evaluate the demographic characteristics of these infections, patient data, limited to sex, age at sample submission, and inpatient/outpatient status, were also collected [10].

### 3.2. Identification of P. aeruginosa Isolates

Some 10 µL of each uncentrifuged urine sample was cultured on UriSelect chromogenic agar (Bio-Rad, Berkeley, CA, USA) and blood agar (bioMérieux, Marcy-l’Étoile, Lyon, France) plates with a calibrated loop, according to the manufacturer’s instructions, and incubated at 37 °C for 24–48 h, aerobically. In the period between 2008 and 2012, presumptive, biochemical reaction-based methods and VITEK 2 Compact ID/AST (bioMérieux, Marcy-l’Étoile, France) were used for bacterial identification. From 2013 onward, MALDI-TOF MS (Bruker Daltonik Gmbh., Billerica, MA, USA) was introduced into the workflow of the Department of Bacteriology. Mass spectrometry was performed using a Microflex MALDI Biotyper (Bruker Daltonics, Bremen, Germany) instrument, with MALDI Biotyper RTC 3.1 software (Bruker Daltonics, Bremen, Germany), and the MALDI Biotyper Library 3.1 for the spectrum analysis. The sample preparation, methodology, and the technical details of the MALDI-TOF MS measurements are described elsewhere [9,10].

### 3.3. Antibiotic Susceptibility Testing and Determination of the Minimum Inhibitory Concentrations (MICs)

Antimicrobial susceptibility testing was performed using the Kirby–Bauer disk diffusion method (Liofilchem, Abruzzo, Italy) on Mueller–Hinton agar (MHA) plates for ceftazidime, cefepime, imipenem, meropenem, ciprofloxacin, levofloxacin, gentamicin, tobramycin and amikacin. MICs of the isolates were determined using E-tests (Liofilchem, Abruzzo, Italy) on MHA plates for ceftazidime, cefepime, imipenem, and meropenem. In addition, for the verification of discrepant results, the broth microdilution method in a cation-adjusted Mueller–Hinton broth (MERLIN Diagnostika, Bonn, Germany) was also utilized. Colistin susceptibility was performed using the broth microdilution method in a cation-adjusted Mueller–Hinton broth (MERLIN Diagnostik). The interpretation of the results was based on the European Committee on Antimicrobial Susceptibility Testing (EUCAST) breakpoints [45]. Car-R/Ceph-S *P. aeruginosa* was defined as isolates resistant to carbapenems (imipenem or meropenem), but susceptible to cephalosporins (ceftazidime or cefepime) [14]. *Staphylococcus aureus* ATCC 29213, *Enterococcus faecalis* ATCC 29212, *Proteus mirabilis* ATCC 35659, *Escherichia coli* ATCC 25922, *P. aeruginosa* ATCC 27853 and *Acinetobacter baumannii* ATCC 19606 were used as quality control strains. Intermediate (I) susceptibility results were not observed during the study. Classification of the isolates as multidrug-resistant (MDR) or extensively drug-resistant (XDR) was based on the EUCAST Expert Rules [46].

### 3.4. Phenotypic Detection of AmpC Overexpression and Carbapenemase Production

Overexpression of AmpC β-lactamase enzymes was detected by an agar plate method, where the agar base was supplemented with cloxacillin (250 µg/mL), as cloxacillin inhibits the effects of AmpC β-lactamases. A two-fold decrease in ceftazidime MICs in the presence of cloxacillin, compared to MICs without cloxacillin, was considered as positivity for AmpC overexpression [14,47]. Phenotypic screening for carbapenemase production was detected by the modified Hodge (cloverleaf) test, optimized for *P. aerugniosa*, as previously described [39,48].

### 3.5. Phenotypic Detection of Efflux Pumps

The effect of phenylalanine-arginine β-naphthylamide (PAβN; a compound with well-known efflux pump inhibitory activity) on the MICs of imipenem and meropenem was detected using the agar dilution method described previously [14,47]. During the experiments, the concentration of PAβN was 40 µg/mL in the agar base. A two-fold decrease in the MICs in the presence of PAβN, compared to the MIC values without the inhibitor, was considered as positivity for efflux pumpoverexpression [14,49,50].

### 3.6. Statistical Anaylsis

Statistical analyses, including the descriptive analysis (means or medians with ranges and percentages to characterize data) and statistical tests (Student’s *t*-test (for data on resistance levels)) were performed with the SPSS software version 24 (IBM SPSS Statistics for Windows 24.0, IBM Corp., Armonk, NY, USA); *p*-values < 0.05 were considered statistically significant.

## 4. Conclusions

The main objective of the present study was the epidemiological assessment and phenotypic characterization of Car-R/Ceph-S *P. aeruginosa* isolates in a tertiary-care hospital in Szeged, Hungary. As a result, *n* = 56 Car-R/Ceph-S *P. aeruginosa* isolates were identified as part of a 10-year surveillance. During the study, overexpression of efflux pumps and overexpression of AmpC enzymes were identified as the main mechanisms of resistance. To spare last-resort agents in relevant clinical situations, the use of broad-spectrum cephalosporins or safe, alternative agents should be considered in these infections to curb the development of extended selection pressure on non-fermenting Gram-negative bacteria, a group of pathogens where the emergence of extensively drug-resistant and pandrug-resistant clinical strains is a frightening reality.
